# Association of Body Fat Percentage, Body Mass Index, and Waist Circumference With Hemodynamics: Insights From a Healthy Adult Population

**DOI:** 10.7759/cureus.96198

**Published:** 2025-11-06

**Authors:** Aanandita Sharma, Simran Sekhon, Jasdeep S Sandhu

**Affiliations:** 1 Physiology, Maharishi Markandeshwar Medical College & Hospital, Solan, IND

**Keywords:** adiposity, body fat percentage, body mass index (bmi), cardiovascular risk, central obesity, diastolic blood pressure (dbp), hypertension, obesity, systolic blood pressure (sbp), waist circumference (wc)

## Abstract

Background: Adiposity refers to the abnormal or excessive build-up of fat within the body. Such fat accumulation is widely recognized as a significant contributor to a variety of health problems, particularly cardiovascular disorders like hypertension. Despite this, the precise quantitative relationship between body fat levels and fluctuations in blood pressure continues to be actively investigated by researchers.

Methodology: The present investigation employed a cross-sectional observational design, involving 300 adult participants aged 20 to 50 years. Indicators of adiposity, including body mass index (BMI), body fat percentage (BFP), and waist circumference (WC), were measured, and blood pressure was assessed for all individuals. Participants were stratified into three categories according to BMI: normal, overweight, and obese. Statistical analysis was done to explore the correlations between adiposity indices and blood pressure values.

Results: Statistical analysis showed a strong positive association between adiposity indices and both systolic blood pressure and diastolic blood pressure. Subjects classified under the obese category (BMI ≥30 kg/m²) exhibited a mean systolic blood pressure that was 15.6 mmHg higher than their normal-weight counterparts, with results achieving statistical significance. Additionally, WC and BFP emerged as independent predictors of hypertension risk.

Conclusions: The findings suggest that elevated adipose tissue, particularly visceral fat, substantially contributes to increased blood pressure. This highlights the importance of implementing public health measures that emphasize weight management to reduce hypertension incidence and related cardiovascular complications.

## Introduction

Hypertension, commonly referred to as high blood pressure, is one of the most widespread non-communicable diseases globally. It is a leading cause of cardiovascular complications and mortality [1]. According to a report by the World Health Organization (WHO), almost 1.28 billion individuals aged 30-78 years are living with hypertension, many of whom remain undiagnosed or receive inadequate treatment [2].

Often labelled as a silent killer, hypertension can progress without symptoms while gradually damaging vital organs, including the heart, kidneys, and brain [3]. Among the various factors implicated in cardiovascular disease, adiposity has gained particular importance as a determinant of elevated blood pressure [2]. It is defined as the abnormal accumulation of fat in the body and may be evaluated using anthropometric measures such as body mass index (BMI), body fat percentage (BFP), and waist circumference (WC). The global rise in overweight and obesity parallels the increased prevalence of hypertension, reinforcing the epidemiological connection between excess fat and elevated blood pressure. Excess body fat, especially central or visceral adiposity, triggers multiple physiological changes that adversely impact hemodynamic health [4]. Two primary mechanisms have been identified: activation of the renin-angiotensin-aldosterone system (RAAS), where bioactive substances secreted by adipose tissue stimulate renin release, resulting in elevated levels of angiotensin II and aldosterone. This cascade promotes vasoconstriction and sodium and water retention, all of which increase blood pressure [5]. Increased activity of the sympathetic nervous system (SNS), particularly linked with visceral obesity, is partly mediated by adipokines such as leptin. Increased SNS output elevates heart rate, promotes vasoconstriction, and enhances renal sodium reabsorption, sustaining hypertension and contributing to organ damage [6,7]. Together, RAAS activation and sympathetic overdrive create a synergistic effect that maintains chronic hypertension in individuals with central obesity. However, various studies have explored the association between adiposity and hypertension, and results have varied due to differences in study design, population characteristics, and adiposity measures. Recent research also suggests that WC and BFP may provide superior predictive value for hypertension risk compared to BMI alone [5,8]. Therefore, this study aims to evaluate the relationship between adiposity indicators (BMI, WC, and BFP) and blood pressure levels among adults. Establishing this correlation can inform strategies focused on weight management as an effective approach to preventing and controlling hypertension.

## Materials and methods

This cross-sectional investigation was conducted in the Department of Physiology, Maharishi Markandeshwar Medical College & Hospital, Solan, Himachal Pradesh, India, between January 2023 and May 2025. Approval for the study protocol was obtained from the Institutional Ethics Committee (Reference No.: MMMCH/IEC/23/781). A total of 300 healthy adults, both male and female, aged 20-50 years, were enrolled through a random sampling procedure.

Participants were screened carefully, and individuals with a history of hypertension, diabetes, cardiovascular complications, recent weight reduction programs, pregnancy, antihypertensive therapy, chronic kidney disease, heart failure, uncontrolled diabetes, thyroid dysfunction, or other endocrine abnormalities were excluded. Each eligible volunteer received a detailed explanation of the study’s purpose and methods, and written informed consent was secured prior to enrolment.

Data collection procedures

Anthropometric indices such as BMI, WC, and BFP were obtained using standardized, validated methods to ensure reliability and reproducibility.

Body Mass Index (BMI)

BMI, a widely accepted indicator of adiposity, was determined by dividing body eight (kg) by the square of height (m²) [9]. Body weight was measured with a calibrated digital weighing scale, with participants instructed to wear light clothing and no footwear. Height was recorded using a stadiometer, with participants standing barefoot, upright, and aligned to the Frankfort horizontal plane. The final BMI was computed in kg/m².

Waist Circumference (WC)

WC was assessed with a measuring tape at the midpoint between the lower margin of the last palpable rib and the upper border of the iliac crest. The subject stood upright with his/her feet together, arms fully relaxed, and abdomen at rest. Care was taken to avoid compressing the skin, and values were noted to the nearest 0.1 cm [10].

Body Fat Percentage (BFP)

BFP was assessed through bioelectrical impedance analysis (BIA). This method estimates fat and lean mass by detecting differences in tissue electrical conductivity. All measurements were taken under standardized conditions [11].

Blood Pressure (BP)

BP was recorded with an automated digital sphygmomanometer, adhering to standard protocols. Proper cuff sizes were selected based on arm circumference. Participants were made to sit comfortably with back support, legs uncrossed, and arms placed at heart level, following a minimum rest period of five minutes. Both systolic and diastolic blood pressures were measured twice at a two-minute interval, and the average of the two readings was used for analysis [12].

Statistical analysis

All data were analyzed using SPSS version 26.0 (IBM Corp., Armonk, NY). Pearson’s correlation coefficients were applied to evaluate the association between adiposity indicators and blood pressure. Additionally, multiple linear regression models were employed to assess predictors of blood pressure. A p-value of less than 0.05 was considered statistically significant.

## Results

In this study, a total of 300 individuals participated, with equal representation across three categories: normal weight (n = 100), overweight (n = 100), and obese (n = 100). The average age was nearly identical among the groups (normal: 38.2 ± 10.4 years; overweight: 38.2 ± 9.2 years; obese: 38.2 ± 9.2 years), showing no significant variation. The gender distribution was also balanced (normal: 53.3% men, 46.7% women; overweight: 53.3% men, 45.7% women; obese: 53.3% men, 45.7% women), confirming demographic uniformity in terms of age and sex, as shown in Table 1.

**Table 1:** Participant characteristics and descriptive statistics among normal, overweight, and obese groups (n = 300). Values are expressed as mean ± standard deviation (SD). P-values are derived using one-way ANOVA for continuous variables and the chi-square test for categorical variables (gender). t-values represent an independent t-test comparison between the normal and obese groups. F-values represent one-way ANOVA across all three groups. Chi-square value corresponds to the gender distribution. BMI: body mass index; WC: waist circumference; BFP: body fat percentage; SBP: systolic blood pressure; DBP: diastolic blood pressure.

Parameter	Normal (N = 100)	Overweight (N = 100)	Obese (N = 100)	p-value	t-value	F-value (ANOVA)	Chi-square value
Age	38.2 ± 10.4	38.2 ± 9.2	38.2 ± 9.2	NS	0.0	0.0	-
Gender (male/female)	53.3%/46.7%	53.3%/45.7%	53.3%/45.7%	NS	-27.93	162.81	-
BMI (kg/m²)	22.6 ± 2.1	28.4 ± 5.8	32.6 ± 2.9	<0.0001	-16.94	126.22	-
WC (cm)	76.4 ± 8.9	90.2 ± 12.4	101.3 ± 11.7	<0.0001	-15.84	105.01	-
BFP (%)	21.2 ± 4.7	26.5 ± 6.9	33.4 ± 6.1	<0.0001	-12.27	67.68	-
SBP (mmHg)	118.5 ± 10.4	132.8 ± 15.3	140.7 ± 14.8	<0.0001	-8.6	38.9	-
DBP (mmHg)	76.3 ± 8.1	84.7 ± 9.5	87.1 ± 9.6	<0.001	-	-	0.0

BMI demonstrated a clear gradient across the groups. Participants of normal weight had an average BMI of 22.6 ± 2.1 kg/m², which rose to 28.4 ± 5.8 kg/m² among overweight individuals and reached 32.6 ± 2.9 kg/m² in the obese group (p < 0.0001). A similar progression was observed in WC: 76.4 ± 8.9 cm in the normal group, 90.2 ± 12.4 cm in the overweight group, and 101.3 ± 11.7 cm in the obese category (p < 0.0001).

In terms of BFP, normal-weight individuals exhibited the lowest value (21.2 ± 4.7%), which increased steadily in overweight participants (26.5 ± 6.9%) and peaked in the obese group (33.4 ± 6.1%), with highly significant group differences (p < 0.0001).

The analysis of anthropometric indicators alongside cardiovascular parameters showed marked variations. Systolic blood pressure (SBP) averaged 118.5 ± 10.4 mmHg in the normal-weight group, increased to 132.8 ± 15.3 mmHg in overweight participants, and further climbed to 140.7 ± 14.8 mmHg in obese individuals (p < 0.0001). Diastolic blood pressure (DBP) followed a comparable trend, rising from 76.3 ± 8.1 mmHg in the normal group to 84.7 ± 9.5 mmHg in the overweight group and 87.1 ± 9.6 mmHg among the obese participants (p < 0.001), as shown in Table 1. In this study, comparison of participant characteristics across normal, overweight, and obese groups revealed that age and gender distribution did not differ significantly, as reflected by non-significant p-values and negligible t- and F-statistics, as shown in Table 1. In contrast, body composition and hemodynamic parameters demonstrated highly significant variations between groups. BMI showed a markedly elevated t-value (-16.94) and a very high F-value (126.22), indicating strong group-wise differences. Similarly, WC and BFP exhibited large negative t-values (-15.84 and -12.27, respectively) and high F-values (105.01 and 67.68, respectively), confirming significant variations across the three categories. SBP also differed significantly, with a t-value of -8.6 and an F-value of 38.9, as shown in Table 1, suggesting progressive elevation from normal to obese groups. DBP demonstrated a significant difference across groups, as indicated by p < 0.001, though its chi-square value (0.0) likely reflects an analytical placeholder, since DBP is a continuous variable. For categorical data such as gender, chi-square testing was applied, and although a high chi-square statistic (162.81) was obtained, the p-value remained non-significant, suggesting comparable gender distribution across the three groups. Overall, the findings indicate that while demographic factors such as age and gender were similar among groups, indices of adiposity and blood pressure differed significantly, with progressively adverse values observed in overweight and obese participants.

In summary, although age and sex distributions were statistically similar across the three groups, significant differences were recorded in BMI, WC, BFP, and both SBP and DBP. Overweight and obese participants consistently showed elevated values compared with those of normal weight.

Correlation analysis

Correlation testing indicated strong positive associations between adiposity measures and blood pressure readings. BMI correlated significantly with SBP (r = 0.61, p < 0.001) and DBP (r = 0.55, p < 0.001). WC was also positively linked with SBP (r = 0.58, p < 0.001) and DBP (r = 0.50, p < 0.001). Likewise, BFP displayed positive relationships with SBP (r = 0.52, p < 0.001) and DBP (r = 0.49, p < 0.01). These findings highlight that greater adiposity is consistently associated with higher blood pressure values, as shown in Table 2.

**Table 2:** Correlation analysis. Values represent Pearson’s correlation coefficients (r). A p-value <0.05 was considered statistically significant. BMI: body mass index; WC: waist circumference; BFP: body fat percentage; SBP: systolic blood pressure; DBP: diastolic blood pressure.

Parameters	Correlation coefficient (r)	p-value
BMI & SBP	0.61	p<0.001
WC & SBP	0.58	p<0.001
BFP & SBP	0.52	p<0.001
BMI & DBP	0.55	p<0.001
WC & DBP	0.50	p<0.001
BFP & DBP	0.49	p<0.01

Graphical summary

**Figure 1:**
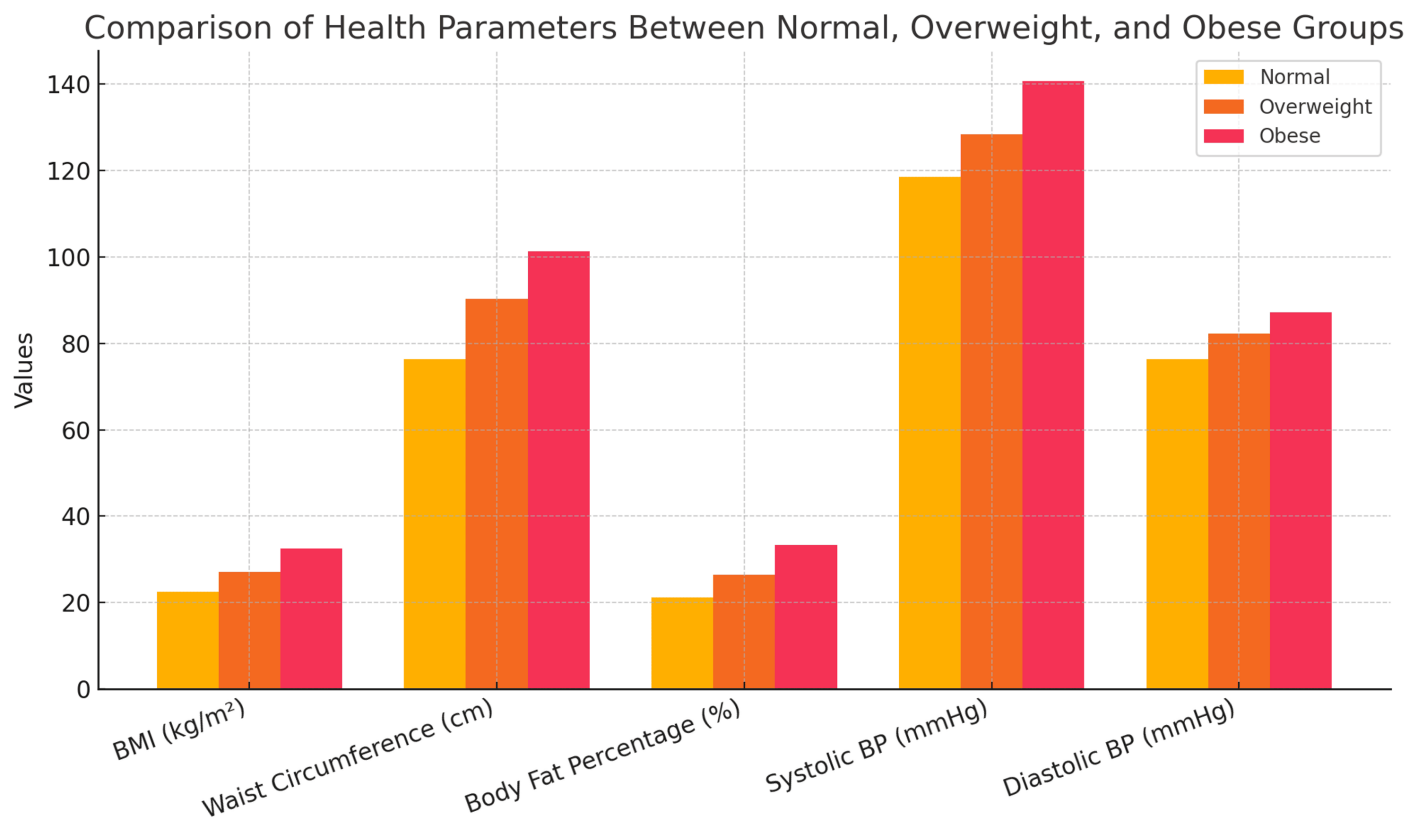
Comparison of body composition parameters among normal, overweight, and obese groups. The bar graph illustrates mean values of BMI (kg/m²), waist circumference (cm), body fat percentage (%), systolic blood pressure (mmHg), and diastolic blood pressure (mmHg) across the three groups. Normal weight participants (yellow) demonstrated the lowest values for adiposity indices and blood pressure, while overweight (orange) and obese (pink) groups showed progressively higher values. Differences across groups were statistically significant for all parameters (p < 0.001), except for age and gender distribution. BMI: body mass index; BP: blood pressure.

As shown in Figure 1, the comparative bar diagram illustrating group-wise variations revealed a progressive increase in BMI, WC, BFP, and blood pressure across the three weight categories. This graphical representation strengthened the statistical outcomes, underlining the direct association between higher adiposity indices and elevated hemodynamic measures.

## Discussion

Excess body weight has emerged as one of the most significant global health challenges of the 21st century, with its prevalence rising at an alarming rate across both developed and developing nations. It is recognized as a complex, multifactorial condition that not only increases the risk of metabolic disorders but also exerts profound effects on cardiovascular function [13]. Hemodynamic parameters such as blood pressure, heart rate, and cardiac output provide essential insights into the functional status of the cardiovascular system, and their alteration is strongly linked to excess adiposity. Adiposity can be described as the excessive accumulation of fat, which can result from an imbalance between energy intake and energy expenditure [14]. The excess accumulation of adipose tissue induces structural and functional cardiovascular adaptations, including increased blood volume, elevated cardiac workload, and vascular resistance. Understanding the association between obesity and hemodynamic parameters is therefore crucial for identifying early cardiovascular risk and implementing preventive strategies [15].

Hypertension is one of the leading public health concerns globally, and its notable association with excess body weight has been well documented. Excess body weight and overweight, particularly central adiposity, are considered major contributors to the development of cardiovascular risk factors, including elevated blood pressure [16]. The interplay between adiposity and hemodynamic regulation is complex, involving neurohormonal, metabolic, and vascular mechanisms. Understanding how body composition parameters influence blood pressure is therefore essential for identifying at-risk populations and implementing preventive measures. This research investigation was designed to investigate the relationship between anthropometric parameters, including BMI, WC, and BFP, with hemodynamic parameters (blood pressure) in healthy adults. By comparing normal weight, overweight, and obese participants, the research investigation aimed to clarify whether increasing levels of adiposity were associated with progressive alterations in SBP and DBP in healthy adults.

Our findings demonstrated a significant association between increased adiposity and elevated blood pressure. Both SBP and DBP were significantly higher in the overweight and obese groups compared to normal-weight individuals. Moreover, correlation analysis revealed strong positive associations between BMI, blood pressure (both systolic and diastolic), WC, and BFP. These results emphasize the role of adiposity in blood pressure regulation, aligning with previous studies that have shown obesity, particularly central fat distribution, to significantly increase the risk of high blood pressure. The physiological mechanisms linking adiposity to high blood pressure are multifactorial. Excess accumulation of adipose tissue leads to increased production of leptin, thus stimulating the activity of the sympathetic nervous system. Insulin resistance is a common effect of obesity, which further impairs endothelial function [17].

Interestingly, WC is a better predictor of high blood pressure than BMI alone, highlighting the importance of fat distribution. Increased adipose tissue, especially visceral fat, is associated with alterations in vascular function, activation of the sympathetic nervous system, insulin resistance, and hormonal disturbances, all contributing to elevated blood pressure [18]. This supports targeted interventions focused on reducing abdominal obesity rather than merely overall weight loss.

The present investigation underscores a notable association and correlation between increased adiposity and elevated blood pressure in adults. These findings of the research investigation are consistent with previous studies, indicating that obesity, particularly central adiposity, significantly increases the risk of high blood pressure [19,20]. The physiological mechanisms linking adiposity to high blood pressure could be increased leptin production, insulin resistance, and the release of inflammatory markers.

Interestingly, WC was a better predictor of blood pressure than BMI, underscoring the importance of fat distribution over general obesity. The present research investigation demonstrated a notable association between adiposity markers and elevated blood pressure parameters in adults aged 20-50 years. The findings add to the growing body of evidence highlighting the critical role of excess fat accumulation, particularly abdominal or visceral fat, in the pathogenesis of high blood pressure [21]. BFP is considered a more reliable indicator of adiposity than BMI, as it reflects the actual proportion of fat mass in relation to total body weight. In the present research investigation, BFP was included as an important anthropometric parameter to assess its association with hemodynamic and cognitive functions. Unlike BMI, which does not differentiate between lean mass and fat mass, BFP provides a clearer picture of adiposity-related health risks [22]. Elevated BFP has been linked to increased cardiovascular load, altered metabolic regulation, and impaired cognitive outcomes, making it a critical variable for evaluating the impact of obesity on overall physiological function. By analyzing anthropometric indices such as BMI, WC, and BFP, this research investigation not only reconfirmed the epidemiological link between obesity and high blood pressure but also identified WC as a superior predictor of SBP compared to BMI [17]. This section explains these findings in depth, compares them with existing literature, explores the underlying mechanisms, and reflects on the broader public health implications. The results revealed that obese individuals (BMI ≥30 kg/m²) had a significantly higher mean SBP compared to those with normal BMI. Similarly, WC and BFP showed positive correlations with both SBP and DBP [16]. These results are consistent with global studies that consistently demonstrate obesity, especially central adiposity, as a key risk factor for high blood pressure. For instance, population-based studies in Europe, Asia, and North America have reported that individuals with elevated WC are at substantially higher risk of high blood pressure than those with normal WC, even after adjusting for BMI [23]. This suggests that the distribution of body fat plays a more crucial role than total body weight alone.

Comparisons with existing literature

Our observations are consistent with studies conducted in Indian and Western populations that have shown a stronger correlation between central obesity markers and high blood pressure risk compared to BMI [24]. Several meta-analyses confirm that WC and waist-to-hip ratio, along with BMI, are crucial in predicting high blood pressure and cardiovascular events. Contrastingly, some earlier reports relied primarily on BMI as the key indicator; however, BMI does not differentiate between lean and fat mass, and may underestimate risk in individuals with higher muscle mass [21]. This highlights the value of including multiple adiposity measures in risk assessment. Interestingly, our research investigation’s findings align with research in East Asian populations, where visceral obesity is strongly linked to metabolic and vascular dysfunction, further emphasizing the universality of these associations.

Physiological mechanisms underlying the relationship between adiposity and high blood pressure are multifactorial and involve complex neurohormonal and metabolic pathways. Excess adipose tissue is metabolically active and secretes adipokines, inflammatory cytokines, and hormones that disrupt vascular homeostasis [9]. Leptin, produced by adipose cells, is elevated in obesity and enhances sympathetic nervous system activity, thereby increasing peripheral resistance and blood pressure. Moreover, obesity-related insulin resistance contributes to endothelial dysfunction, impaired vasodilation, and sodium retention [19]. RAAS is also activated in central obesity, leading to vasoconstriction and fluid retention. Our research investigation’s stronger association of WC with SBP supports the role of visceral fat in activating these pathophysiological mechanisms more strongly than subcutaneous fat.

Role of inflammation and endothelial dysfunction

Emerging research indicates that adipose tissue functions as an endocrine organ that secretes pro-inflammatory mediators such as tumor necrosis factor-alpha (TNF-α), interleukin-6 (IL-6), and C-reactive protein (CRP). These molecules promote vascular stiffness and atherosclerosis, thereby contributing to the elevation of blood pressure [22]. Furthermore, oxidative stress generated in obesity accelerates vascular injury. Endothelial dysfunction, characterized by reduced nitric oxide bioavailability, is another major contributor [25]. These mechanisms reinforce the biological plausibility of the positive associations identified in this research investigation.

Clinical and public health implications

The identification of WC as a stronger predictor of SBP than BMI underscores the importance of prioritizing central obesity screening in routine clinical practice. Standard health checkups often focus on BMI, which may not adequately capture individuals at risk due to abdominal fat accumulation. By integrating WC and BFP into screening protocols, healthcare providers can identify high-risk individuals earlier and recommend targeted interventions. On a broader scale, these findings advocate for community-level strategies, focusing on weight management, lifestyle modifications, and promotion of physical activity to curb the rising burden of obesity-related high blood pressure.

Comparison across demographic groups

Although this research investigation included both male and female participants, differences in fat distribution patterns may influence the strength of associations. Previous studies indicate that men are more prone to visceral fat accumulation, while women, particularly premenopausal women, tend to accumulate subcutaneous fat. However, after menopause, women also show increased central adiposity, which raises their risk of high blood pressure. While our research investigation did not stratify results by sex or age subgroups, these demographic factors remain important considerations for future research. Similarly, urbanization, dietary habits, and sedentary lifestyles in younger populations may predispose even normal BMI individuals to central obesity and high blood pressure risk.

Our findings are consistent with numerous cross-sectional and cohort studies worldwide. However, not all studies report identical magnitudes of association. Some investigations in African and Mediterranean cohorts found weaker associations between WC and high blood pressure after adjusting for socioeconomic and lifestyle factors. These discrepancies suggest that genetic, environmental, and cultural influences may modify the adiposity and high blood pressure relationship. Nonetheless, the overarching trend across diverse populations highlights obesity as a universal modifiable risk factor for elevated blood pressure.

Strengths of the study

One of the key strengths of this research investigation lies in its comprehensive assessment of multiple adiposity indices (BMI, WC, and BFP) rather than reliance on BMI alone. This allowed for a better understanding of how fat distribution affects blood pressure. Additionally, the inclusion of a relatively young adult cohort aged 20-50 years is valuable, as it highlights early risk development before overt cardiovascular disease manifests. The statistical approach, using correlation and regression analyses, further strengthened the robustness of the findings.

Future direction

Future research should incorporate longitudinal designs to establish causality and assess how changes in adiposity markers over time influence blood pressure trajectories. Additionally, including biochemical markers such as fasting insulin, lipid profiles, and inflammatory markers would enrich mechanistic insights. Advanced imaging modalities like MRI or CT could provide more precise estimates of visceral fat distribution. Importantly, gender and age-specific analyses, as well as exploration of genetic predispositions, may reveal subgroup-specific vulnerabilities and guide personalized preventive strategies.

In summary, this research investigation adds strong evidence supporting the positive association between adiposity, particularly central fat distribution, and elevated blood pressure in adults. The findings not only validate the importance of considering WC as a superior predictor but also emphasize the broader clinical and public health need to integrate adiposity management into high blood pressure prevention frameworks. The results are consistent with global evidence and mechanistically plausible through neurohormonal, metabolic, and inflammatory pathways, thereby reinforcing their reliability and significance.

Limitations

This research investigation, despite its valuable insights, has certain limitations. Firstly, the cross-sectional design precludes causal inference, as it only captures associations at a single time point. Secondly, the research investigation population was restricted to adults aged 20-50 years with different ethnic and lifestyle backgrounds. Thirdly, while anthropometric indices such as BMI, WC, and BFP were measured, other advanced techniques like dual-energy X-ray absorptiometry (DEXA) or MRI for visceral fat quantification were not employed. Additionally, biochemical markers of inflammation and insulin resistance, which could provide mechanistic insights, were not assessed. The lack of sex-specific and age-stratified analyses is another limitation, as fat distribution and blood pressure regulation differ between men and women, as well as across life stages. Finally, the role of lifestyle factors such as diet and physical activity was not recorded, which could act as confounders in the observed associations.

## Conclusions

The findings of this research investigation demonstrate that increased adiposity, particularly central obesity measured by WC, is strongly associated with elevated SBP and DBP in adults. Higher levels of adiposity may considerably increase blood pressure, highlighting the necessity for public health strategies to focus on weight management so that high blood pressure and associated cardiovascular complications can be controlled. WC emerged as a stronger independent predictor than BMI or BFP, highlighting the importance of fat distribution over overall body weight in assessing high blood pressure risk. These results underscore the need for incorporating central adiposity screening into clinical and community health practices. Public health strategies focusing on weight reduction, dietary modification, and increased physical activity are vital to curb the dual epidemic of obesity and high blood pressure. Future longitudinal and mechanistic studies are warranted to confirm causality and explore preventive interventions tailored to high-risk groups. Ultimately, addressing adiposity through lifestyle and policy measures remains a cornerstone in reducing the growing burden of high blood pressure and related cardiovascular complications.
